# Distributed Fiber-Optic Shape Sensing with Endpoint Error Compensation: Theory and Experimental Validation

**DOI:** 10.3390/s26072156

**Published:** 2026-03-31

**Authors:** Leonardo Rossi, Francesco Falcetelli, Francesco Gagliardo, Piero Lovato, Filippo Bastianini, Raffaella Di Sante, Gabriele Bolognini

**Affiliations:** 1 Consiglio Nazionale delle Ricerche, 40129 Bologna, Italy; leonardo.rossi@cnr.it (L.R.);; 2 Department of Industrial Engineering—DIN, University of Bologna, 47121 Forlì, Italy; francesco.gagliardo@studio.unibo.it (F.G.); raffaella.disante@unibo.it (R.D.S.); 3 Socotec Monitoring Italy S.r.l., Via Torretta, 20/C, Calderara di Reno, 40012 Bologna, Italy; piero.lovato@socotec.com (P.L.); filippo.bastianini@socotec.com (F.B.)

**Keywords:** optical fiber sensors, BOTDA, shape sensing, sensing cable, calibration, optimization, error compensation algorithm

## Abstract

Fiber-optic shape sensing enables real-time monitoring of structural deformation across a wide range of applications. For large-scale structures, Brillouin-based distributed sensing, typically implemented through Brillouin Optical Time Domain Analysis (BOTDA), offers an extended range for quasi-static measurements, albeit its limited spatial resolution degrades reconstruction accuracy. This study addresses this fundamental limitation through the introduction of a novel error compensation algorithm, particularly suited for a Brillouin-based shape sensing system, yet agnostic with respect to the sensing technology. The method leverages both the initial and final points of the sensing path, performing both forward and backward reconstructions and fusing the two trajectories by testing several polynomial and exponential weighting strategies. The algorithm is experimentally validated on a 28.91 m four-core shape sensing fiber cable (length = L), interrogated through BOTDA operating at 50 cm spatial resolution, and reconstructed through the Frenet–Serret frame formulation. Calibration procedures include radial-offset tuning and segment alignment via a hotspot reference. A non-trivial S-shaped geometry is adopted as a case study, specifically addressing curvature discontinuities arising from mixed straight and curved segments. Reconstruction accuracy is quantified through a Euclidean-distance-based Figure of Merit (FOMs). The cubic weighting strategy demonstrates improvements exceeding 86% in all FOMs compared to classical methods without compensation. Specifically, it achieves an RMSE of 0.145 m (0.50% of L), a MAE of 0.109 m (0.38% of L), and a maximum error of 0.341 m (1.18% of L). Remarkably, these percentage errors are of the same order of magnitude as those reported in the literature for Fiber Bragg Grating (FBG) and Optical Frequency Domain Reflectometry (OFDR) systems, indicating that the proposed compensation strategy enables BOTDA-based shape sensing to achieve comparable reconstruction accuracy despite its lower spatial resolution.

## 1. Introduction

Optical fiber sensors (OFS) offer several advantages that have led to their widespread adoption across a broad range of applications. In the field of Structural Health Monitoring (SHM), OFS is extensively used for damage or vibration detection in various types of infrastructure [1,2,3,4,5].

In some cases, knowledge of the structural shape can be more informative than the strain field alone or can provide complementary information. This has motivated the development of shape reconstruction methodologies. Although shape reconstruction is not exclusive to optical fiber sensing and can also be achieved using other technologies, OFS remains particularly well-suited for this purpose due to its embeddability [6], small size [7], passive operation [8] and long sensing range [9], which can also be further improved with distributed amplification techniques, such as Raman amplification, which can allow for lightwave signal amplification up to several tens of kms in higher-order schemes [10].

Over the past decade, this research area has been highly active, resulting in substantial scientific literature addressing diverse applications, novel reconstruction algorithms, and dedicated shape-sensing cable designs. Comprehensive reviews of OFS-based shape sensing have been provided by Floris et al. [11], Amanzadeh et al. [12], and more recently by Zhai et al. [13].

Shape sensing can be implemented by performing strain measurements either through a multicore optical fiber or a bundle of single-core fibers within an optical fiber cable. The multicore approach results in compact shape sensing cables but comes at the cost of requiring complex and costly fan-in and fan-out components [14,15]. In contrast, fiber bundles assembled from standard single-core fibers provide robust mechanical protection and improved curvature sensitivity due to the greater core-to-neutral axis separation, making them suitable for large-scale structural monitoring and, more generally, for applications requiring high curvature resolution [11], albeit at the expense of increased size, limiting their use in space-constrained applications.

**Figure 1:**
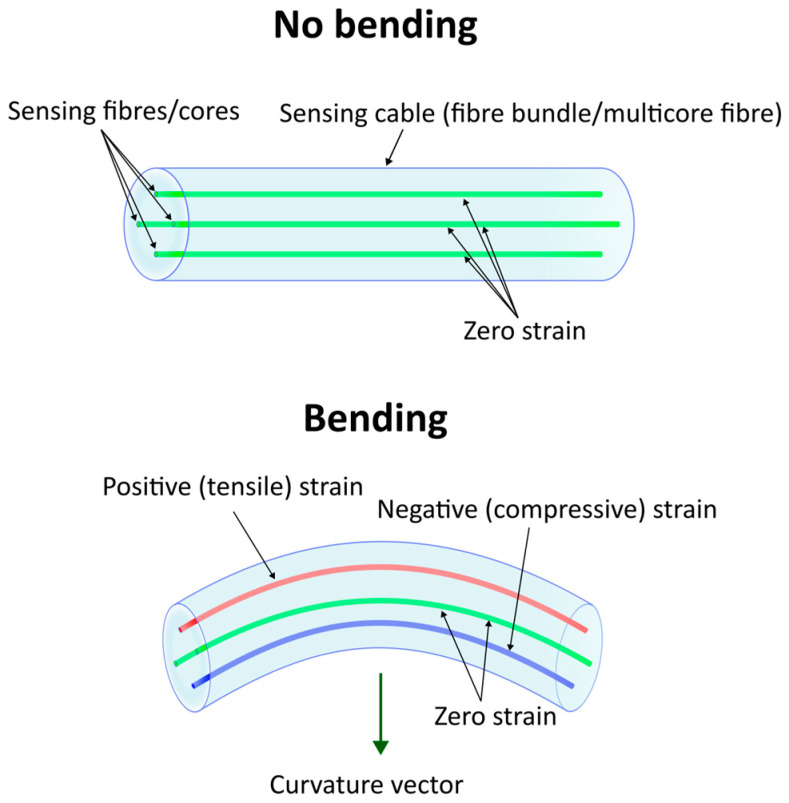
Example of how a shape-sensing cable (be it a fiber bundle or a multicore fiber) works: depending on the way it is bent, its sensing cores in off-center positions will be subject to different strains. By measuring them, the bending vector can be determined.

The way shape sensing can be performed by measuring the strain in cores that are offset from the center of the sensing cable (or multicore fiber) is shown in Figure 1: in the presence of bending, each core will face a different strain depending on its location with respect to the center. By combining the strain measurements of the cores, the bending, which is represented by the curvature vector, can be measured at every point of the sensing cable. From the curvature vectors along the cable, it is possible, for instance, through the Frenet–Serret equations, to calculate the tangent of the curve at every sensing point, which can then be integrated to obtain the complete 3D shape of the sensing cable. A more thorough description will be presented in Section 2.2.

This approach is affected by an issue that has so far remained relatively unexplored: the uncertainty of the 3D position of each sensing point depends both on the measurement uncertainty and the position of the previous point. As a result, the uncertainty builds up, degrading measurement accuracy along the sensing cable [16,17,18].

The prevalence of this issue depends on both the measurement uncertainty (which is linked to both strain accuracy and spatial resolution) and the number of sensing points. In FBG-based systems, the number of sensing points is inherently determined by the number of gratings and their spacing. For instance, Bronnikov et al. and Khan et al. [19,20] employ 6 and 8 FBG triplets with grating spacing of 14 mm and 30 mm, respectively. In terms of accuracy, assuming a typical wavelength stability of 1 pm at 1500 nm and an effective photo-elastic constant of 0.22, the resulting strain uncertainty is on the order of 1 µε. These spatial resolution and strain accuracy values together lead to relatively low shape-reconstruction errors (maximum mean absolute. error lower than 2 mm over a sensing range of 72 mm [19]).

For Optical Frequency Domain Reflectometry (OFDR) based systems, Yin et al. [21] report a sensing-point spacing of 9.4 mm, a 47 mm spatial resolution, and a fiber length of 1200 mm. Meng et al. [22] achieve a spacing of 10.1 mm over a 200 mm multicore fiber. Combined with the low strain uncertainty of OFDR (e.g., 3 µε for standard SMF-28 fibers [23]), these parameters enable accurate shape reconstruction, with end-point RMSE values of approximately 12 mm over 200 mm [22].

These quantitative values highlight that both FBG- and OFDR-based approaches can mitigate error propagation in shape sensing. Still, their sensing range is usually limited.

In the case of FBGs, the number of gratings that can be inserted in a sensing fiber is limited. Considering the examples of [19,20], in both cases, the achievable sensing range was less than 1 m. While this range could be extended by increasing FBG spacing, this would, of course, come at the cost of poorer spatial resolution.

In the case of OFDR applied to shape sensing, outside of the already cited examples from [21] and [22], where the measurement range was 1200 and 200 mm, respectively, a recent review by Ding et al. (2023) also reports measurement ranges of approximately 2–3 m, primarily in medical-related applications [24].

Indeed, it is a general rule of distributed optical fiber sensors that high spatial resolution often comes at the cost of sensing range and vice versa [25,26,27]. This issue limits the use of the sensing techniques mentioned above for applications where longer sensing ranges are required, such as monitoring of both civil and energy infrastructures, such as bridges, skyscrapers, wind turbines, or pipelines.

A distributed optical fiber sensing technology that can allow the acquisition of distributed strain measurements at distances of up to 100 km is Brillouin Optical Time Domain Analysis (BOTDA). This technique has been effectively employed for large structure monitoring and provides additional benefits, such as high dynamic range and the ability to provide temperature alongside strain measurement with a single interrogating device, which are both desirable traits in any potential large-scale shape sensing application.

BOTDA systems generally provide a long measurement range at the cost of reduced spatial resolution and lower strain-measurement accuracy, since the minimum achievable resolution is ultimately limited by the phonon lifetime. Quantitative data in the literature confirm this behavior: Xie et al. report spatial resolutions ranging from 0.05 m to 150 m and strain accuracies in the 20–60 µε interval [28]. These values are significantly lower than those of the FBG-based systems discussed earlier (grating spacing of 30–72 mm [19,20] and typical strain uncertainty of ~1 µε) and of OFDR-based approaches, which achieve ~10 mm sampling intervals and strain uncertainties below 5 µε [23]. When these performance characteristics are combined with the inherently large number of sensing points arising from BOTDA kilometer-scale range, the cumulative error build-up described above becomes particularly critical, making BOTDA-based configurations typically poorly suited for accurate 3D shape reconstruction.

Indeed, within the BOTDA framework, both standard and differential pulse-width-pair (DPP) implementations have been mainly employed as curvature detectors [29]; however, when moving from curvature estimation to full shape sensing, reported demonstrations are limited to DPP-BOTDA implementations, which allow for better spatial resolution (down to a few centimeters), and to simple geometrical configurations [30,31,32].

In this work, we present a novel method to compensate for the error build-up by taking advantage of information on the positions of both the start and end points of the sensing cable. While most shape sensing applications rely on the starting point as the sole known constraint, some scenarios also allow the cable endpoint to be considered as known, particularly in several cases common in real-life applications when the structure’s far end is physically constrained (e.g., a bridge anchorage or a pipeline termination) or can be independently measured using an external instrument such as theodolites [33], linear potentiometers [34,35], or GPS (Global Positioning System) receivers [36]. When this information is available, the 3D shape can be reconstructed in both directions and combined into a single curve with a weighted sum, greatly suppressing the increase in uncertainty.

This approach would enable BOTDA-based shape sensing to be applied to large-scale civil and energetic infrastructure, where it currently fails due to its excessive error accumulation. In particular, it would be suitable for civil infrastructures such as bridges, tunnels, rails, skyscrapers, landslides, and key energetic infrastructures such as wind turbines, dams, pipelines, and large tanks, where the 3D shape must be retrieved over a long sensing range. At present, these structures are typically monitored through sparse sensor networks such as accelerometers, inclinometers, and Global Navigation Satellite System (GNSS) receivers [37], acoustic emissions, or point-based strain sensors, including electrical strain gauges or FBG arrays [38]. Although these technologies represent a set of valuable tools for SHM, they cannot reconstruct the full 3D geometry of the structure. Therefore, this contribution aims to make a significant step forward by enabling long-range 3D shape reconstruction in such applications.

As a showcase of the effectiveness of this technique approach, we present an experiment where we use the Frenet–Serret equations to fully reconstruct the 3D shape of a non-trivial curve (S-shaped trajectory) using a BOTDA sensor with a spatial resolution of 50 cm to detect strain along a 30 m long four-core shape-sensing cable. The S-shaped trajectory was chosen not to mimic a geometrical pattern typical of a specific structure or application, but rather because it provides a more informative benchmark than the simple geometries typically used in shape sensing studies, such as circles [39], helices [19], or single-arc curves [40]. An S-trajectory includes three curvature discontinuities, arising at the transitions between straight and curved regions, which represent a particularly demanding condition for a system with 50 cm spatial resolution due to the associated broadening of the curvature profile. In addition, the presence of long straight segments, where the signal-to-noise ratio is lowest, further stresses the reconstruction process. Overall, this S-shape enables a comprehensive assessment of reconstruction robustness across mixed straight–arc patterns, and, although not intended to reproduce any specific structural asset, it may still be representative of realistic deformation scenarios such as those encountered in large-scale civil infrastructures (e.g., pipeline or tunnel deviations or rail transition curves).

We consider standard Figures of Merit (FOMs) and show that with our approach, the deviation from the ground truth is greatly reduced (>86% for each FOM), showing both the effectiveness of our approach and that BOTDA shape sensors are affected by the error build-up. With this method, a full 3D reconstruction of a curve using a common BOTDA sensor interrogator is showcased for the first time to the authors’ knowledge.

We also include a clear and repeatable calibration procedure for the purposes of both correctly aligning the internal cores and estimating their effective eccentricity, and we employ an off-the-shelf single-channel BOTDA interrogator.

The paper is organized as follows: Section 2 introduces the principles of BOTDA-based shape sensing and outlines the Frenet–Serret framework used for reconstruction. Section 3 presents the proposed error compensation algorithm, including bidirectional reconstruction, weighted fusion, and the design of the weighting function. Section 4 describes the experimental setup and the calibration procedures. Section 5 reports the S-shaped case study and the associated performance evaluation metrics. Section 6 discusses the uncompensated and compensated reconstruction results. Section 7 provides a broader discussion of the methodological contributions, limitations, and implications. Finally, Section 8 concludes the work.

## 2. Principles of BOTDA-Based Shape Sensing

The section introduces both the principles of BOTDA sensing as well as the principles of shape sensing, showing how strain reading inside a sensing cable can be used to reconstruct its 3D structure through the use of the Frenet–Serret equations.

### 2.1. BOTDA

Generally speaking, BOTDA sensing methods involve monitoring how a pulsed signal (pump) amplifies a frequency-downshifted, counter-propagating continuous wave (probe) signal as it travels along the fiber, through a process known as Stimulated Brillouin Scattering (SBS). This process, which originates from the interaction between acoustic phonons in the medium and the electrostriction caused by the beating of the two light waves, has a gain that depends on the pump-probe frequency shift by a Lorentzian relationship, known as the Brillouin Gain Spectrum (BGS). The frequency at which the maximum gain is achieved is known as Brillouin Frequency Shift (BFS) and depends on the speed of sound in the section of fiber where the measurement takes place, which in turn is affected by both temperature and strain. As a consequence, by measuring the amplification of the probe signal at different pump-probe frequency shifts, it is possible to reconstruct the BGS at every point in the fiber, from which the BFS can be extracted through Lorentzian curve fitting [41].

**Figure 2:**
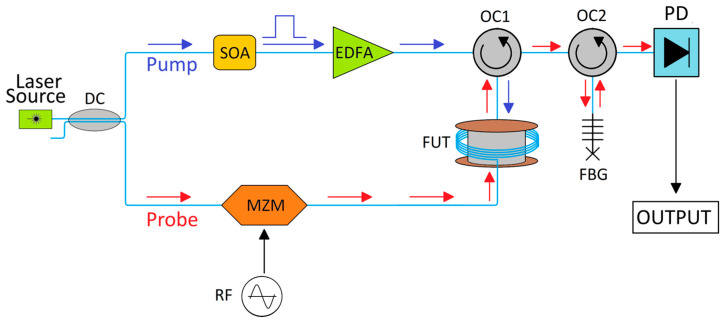
Schematic representation of a BOTDA system based on the double sideband modulation scheme. The blue arrow represents the path of the pulsed pump, while the red arrow represents the path of the CW probe. DC: Directional coupler, SOA: Semiconductor Optical Amplifier, MZM: Mach-Zehnder Modulator, EDFA: Erbium Doped Fiber Amplifier, OC: Optical Circulator, FUT: Fiber Under Test, FBG: Fiber Bragg Grating, PD: Photodetector.

In the following experimental demonstration, we have employed the BOTDA module of the off-the-shelf OZ Optics Foresight™ Distributed Strain and Temperature Sensor (DSTS) [42]. To exemplify how BOTDA sensing typically works, a schematic representation of the sensing principle based on double sideband modulation [34] is shown in Figure 2.

A laser source, usually a distributed feedback (DFB) laser with a wavelength of 1550 nm, is split into a pump and a probe branch by a directional coupler (DC). In the pump branch, the light is shaped into a pulse, for instance by a Semiconductor Optical Amplifier (SOA), and then amplified by an Erbium Doped Fiber Amplifier (EDFA), and then sent into the fiber under test (FUT) through an optical circulator (OC1). On the probe branch, the laser carrier wave is split into two sidebands by a Radio Frequency (RF) modulated Mach-Zehnder modulator (MZM) and sent into the FUT. The two sidebands are respectively upshifted and downshifted by a frequency amount equal to the RF frequency with respect to the pump light. As the probe is sent into the FUT, the downshifted sideband is amplified by the pump through SBS, exiting it through OC1. The upshifted sideband and carrier wave are filtered out by a combination of another optical circulator (OC2) and an FBG, which only reflects the downshifted sideband back into OC2 and into the rest of the setup. The filtered probe is then acquired through a photodetector (PD). By changing the RF modulation frequency, different pump-probe frequency shifts can be evaluated, and the BFS for each point in the fiber can be found.

### 2.2. Frenet–Serret Framework for Shape Reconstruction

Among the various algorithms available for shape sensing, the formulation based on the Frenet–Serret frame is one of the most widely adopted. As highlighted by R. L. Bishop in his seminal paper “There is more than one way to frame a curve” [43]. Alternative formulations, such as the parallel transport frame (Bishop frame) or rotation-minimizing frames (RMF), also exist and can be advantageous in certain situations. In this study, however, we rely on the classical Frenet–Serret frame to keep the mathematical treatment as simple as possible and to demonstrate that, for BOTDA-based sensing, it remains an adequate choice even in the presence of apparent curvature discontinuities.

**Figure 3:**
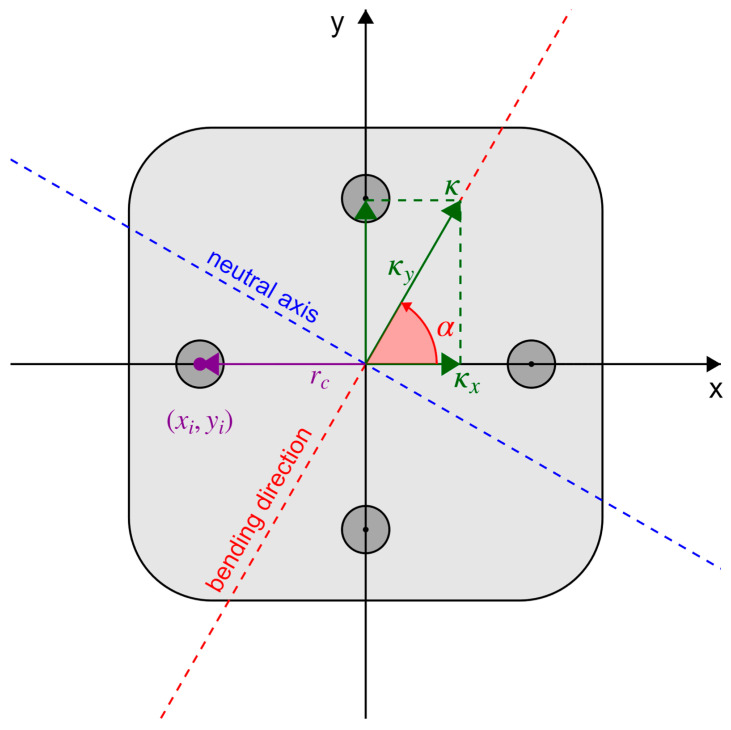
Conceptual cross-section of a 4-core fiber bundle with a bending defined by the curvature vector κ (green), decoupled into its components $\kappa_{x}$ and $\kappa_{y}$ in the local reference frame defined by the axes x and y. The vector direction defines the bending direction, indicated by the red dashed line. Perpendicular to the bending direction is the neutral axis (blue dashed line), along which the bending-induced strain is zero.

Figure 3 shows a simplified axially symmetric four-core fiber bundle used for theoretical explanation, while the actual cable geometry is presented in Section 4.1.

The neutral axis (blue dashed line) and the bending direction (red dashed line) are respectively perpendicular and parallel to the curvature vector κ, which is indicated in the figure by the green arrow. Depending on its magnitude $\left|\kappa \right|$ and orientation α with respect to the cable cross-section reference frame, a specific strain is induced in each core. A larger radial offset $r_{c}$ corresponds to a greater distance from the neutral axis and results in increased bending sensitivity of the sensing cable. The strain measured at the i-th sensing core, located at coordinates $\left(x_{i},\;y_{i}\right)$, denoted as $\varepsilon_{i}$, is given by Equation (1):

(1) $$\varepsilon_{i}=\varepsilon_{long}+\kappa_{x}x_{i}+\kappa_{y}y_{i}$$

Here, $\varepsilon_{long}$ is the longitudinal strain experienced by the sensing cable, and $\kappa_{x}$ and $\kappa_{y}$ are the x- and y-components of the curvature vector, respectively.

Applying Equation (1) to each sensing core leads to a system of algebraic equations which can be solved to find $\varepsilon_{long}$, $\kappa_{x}$, and $\kappa_{y}$.

From the two curvature components, the curvature magnitude $\left|\kappa \right|$ and its orientation α can be computed, followed by the torsion τ, representing the rate of change in the bending angle with respect to the arc length coordinate s. Considering r(s) as the curve position vector (bold symbols denote vector quantities) in its arc-length parametrization, with $r:\left[0,L\right]\to R^{3}$ and L being the total curve length, then it is possible to define the tangent unit vector, T(s), which is always pointing in the curve direction, as:

(2) $$T\left(s\right)=\frac{dr\left(s\right)}{ds}$$

Then, the normal unit vector, N(s), whose orientation matches the curvature direction, is defined as follows:

(3) $$N\left(s\right)=\frac{\frac{dT}{ds}}{\left\|\frac{dT}{ds}\right\|}$$

Finally, we define the binormal unit vector, B(s), which is orthogonal to both $T\left(s\right)$ and $N\left(s\right)$, and $N\left(s\right)$, as the vectorial product:

(4) $$B\left(s\right)=T\left(s\right)\times N\left(s\right)$$

These three vectors constitute the TNB frame (or Frenet–Serret frame) and satisfy the following system of first-order homogeneous linear ordinary differential equations:

(5) $$\left[\begin{array}{c} T\prime \left(s\right)\\ N\prime \left(s\right)\\ B\prime \left(s\right) \end{array}\right]=\left[\begin{array}{ccc} 0 & \kappa \left(s\right) & 0\\ -\kappa \left(s\right) & 0 & \tau \left(s\right)\\ 0 & -\tau \left(s\right) & 0 \end{array}\right]\left[\begin{array}{c} T\left(s\right)\\ N\left(s\right)\\ B\left(s\right) \end{array}\right]$$

**Figure 4:**
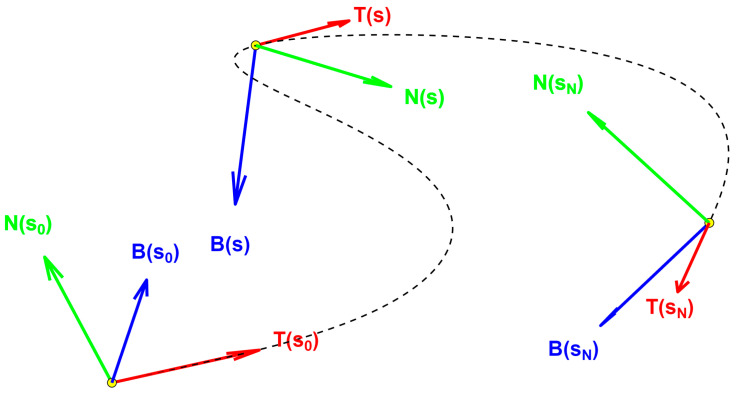
Example of how the Frenet–Serret (TNB) frame moves rigidly along a generic curve.

Equation (5) is also known as the Frenet–Serret formulas expressed as a matrix equation. By definition, the TNB frame forms an orthonormal basis of $R^{3}$, providing an orthonormal moving frame along the three-dimensional curve, as illustrated in Figure 4.

## 3. Error Compensation Algorithm

**Figure 5:**
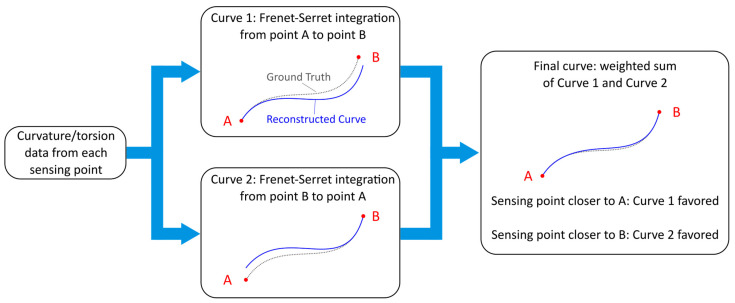
Schematic representation of the bidirectional shape reconstruction principle. Starting from the same curvature and torsion data, the curve is reconstructed first using “A” as a starting point (forward direction), then using “B” (backward direction). The final output is a weighted combination of these two curves.

The section introduces the working principle of the proposed error compensation algorithm. We first outline the core idea underlying the method, which leverages a double forward–backward reconstruction enabled by the knowledge of both the initial and final points of the trajectory (as illustrated in Figure 5). Then, we analyze the fusion of the two reconstructed curves obtained through this procedure, compare different weighting strategies, and finally describe the weighting function itself, which is defined using polynomial or exponential formulations.

### 3.1. Bidirectional Shape Reconstruction

The core idea of this method is to exploit information on the position of both the starting and end points of the sensing cable to reduce overall positional error along the curve.

The procedure consists of three main steps reported in Figure 5. First, the curve is reconstructed using the conventional method based on the Frenet–Serret framework. Second, the same procedure is repeated, but this time using the known ending point as the new starting point. At this stage, we obtain two reconstructed curves derived from the same dataset.

The third step involves merging these two curves into a single, improved curve. The underlying rationale is that reconstruction error tends to increase with distance from the starting point. Therefore, we compute a weighted average of the two curves’ coordinates, assigning greater weight to points closer to their respective starting points. This raises the question of how to define the weighting function, whether it should vary linearly, quadratically, cubically, exponentially, and so forth.

### 3.2. Weighted Curve Fusion

In the bidirectional shape reconstruction approach, the forward and backward reconstructed curves can be defined, respectively, as curve coordinate vectors $r_{f}\left(i\right)$ and $r_{b}\left(j\right)$ ($r_{f},r_{b}\in R^{n\times 3}$), where i=1,…,n represents the coordinate index along the sensing cable, whereas j=n−i+1 denotes the mirrored index for the backward reconstruction.

The final curve $r_{best}\left(i\right)$ is a weighted sum of $r_{f}\left(i\right)$ and $r_{b}\left(j\right)$, and it is defined at the i-th index as:

(6) $$r_{best}\left(i\right)=\frac{w_{f}\left(\xi \right)r_{f}\left(i\right)+w_{b}\left(\xi \right)r_{b}\left(j\right)}{w_{f}\left(\xi \right)+w_{b}\left(\xi \right)}$$

where $w_{f}\left(\xi \right)$ and $w_{b}\left(\xi \right)$ are the forward and backwards weights, respectively, and $\xi \in \left[0,\;1\right]$ is a normalized curvilinear coordinate, defined as:

(7) $$\xi =\frac{i-1}{n-1}$$

### 3.3. Weighting Function

The choice of the weighting function has a direct impact on the reconstruction accuracy of the target shape. Ideally, this function should be determined from the relationship between positional error and noise. As was shown in previous work, which reported error as a function of position (both relative [32] and absolute [16,44]), this relationship is not simple and follows a very complex behavior depending on a variety of curve-specific parameters.

In this work, we decided to test several weighting profiles, such as linear, quadratic, cubic, polynomial (4th and 5th), and exponential, to see how different cost functions will affect the final shape reconstruction error.

The cost functions will be defined as follows. The forward and backward weights are symmetrically defined with respect to the normalized distance ξ parameter as:

(8) $$w_{f}\left(\xi \right)=f\left(1-\xi \right);\;\;w_{b}\left(\xi \right)=f\left(\xi \right),$$

with $f\left(\cdot \right)$ being a monotonically increasing function over the domain $\left[0,\;1\right]$. This definition ensures that $w_{f}$ and $w_{b}$ behave symmetrically, with $w_{f}$ monotonically decreasing from 1 to 0 and $w_{b}$ monotonically increasing from 0 to 1.

For the polynomial case, we can define $f\left(\xi \right)$ as:

(9) $$f\left(\xi \right)=\xi^{k},\;\;k=1,2,3,4,5\;$$

On the other hand, for the exponential case, it is possible to define $f\left(\xi \right)$ as:

(10) $$f\left(\xi \right)=\frac{exp\left(\alpha \xi \right)-1}{exp\left(\alpha \right)-1}\;$$

For comparison purposes, an equivalence between polynomial and exponential weighting functions was also established by matching their midpoint values at ξ=0.5:

(11) $${\left.\xi^{k}\right|}_{\xi =0.5}={\left.\frac{exp\left(\alpha \xi \right)-1}{exp\left(\alpha \right)-1}\right|}_{\xi =0.5}\;$$

Therefore, solving Equation (11) the correspondence between α and k is given by:

(12) $$\alpha =2\ln\left(2^{k}-1\right)$$

This formulation leads to the relationship given in Table 1.

## 4. Experimental Setup and Calibration

The section presents the shape-sensing cable adopted in this study, providing its main material and geometrical characteristics. The description then moves to the BOTDA interrogator settings and the methodology for the baseline acquisition. Attention is devoted to the procedure adopted to ensure that strain samples corresponding to the same arc length positions, yet belonging to different fiber cores, are correctly matched during post-processing; this is achieved through a dedicated hotspot-based alignment method. The section concludes with the radial offset calibration, which assesses the distance of each fiber core from the cable neutral axis by winding the cable around rigid circular supports of known diameter.

### 4.1. Shape Sensing Cable

**Figure 6:**
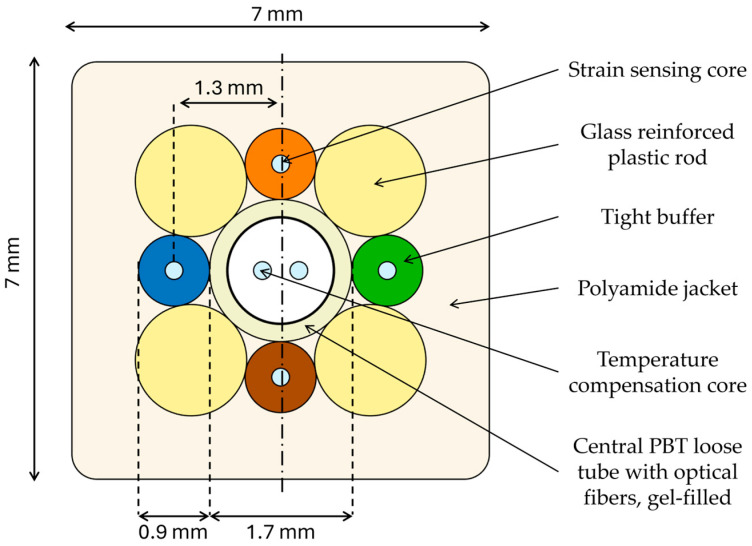
Section of the four-core shape sensing cable employed in this study, highlighting both the fiber cores (in light blue) as well as the main structural elements, including the GRP reinforcing rods, the gel-filled PTB tube, and the external PA jacket.

The optical fiber cable used in this study (manufactured by Socotec Monitoring Italy S.R.L., Bologna, Italy) is shown in Figure 6 (component dimensions not to scale).

The cable is 28.91 m long, has a square cross-section (7 mm × 7 mm), and incorporates four tight-buffered strain-sensing optical fiber cores positioned at the east (green), north (orange), west (blue), and south (brown) locations, each with a radial offset of 1.3 mm from the center. This offset is a key design parameter, as it directly determines the cable’s sensitivity to curvature. The fibers used in the shape-sensing cores comply with ITU-TG.657.A1; for the specific fiber employed in our experiments, the manufacturer specifies a core diameter of 9 μm and a cladding diameter of 125 μm. Four glass-reinforced plastic (GRP) rods located at 45°, 135°, 225°, and 315° provide structural stiffness and help minimize torsional deformation. An inner plastic tube buffer (PTB), filled with gel, reduces friction between the temperature-compensation fibers and the surrounding PTB surface. All components are held in place by a polyamide (PA) jacket. The PA jacket stabilizes the internal structure and protects the cable from harsh environmental conditions. The jacket features a structured surface designed to enhance mechanical coupling with the host structure.

### 4.2. BOTDA Interrogator Setup and Baseline Acquisition

**Figure 7:**
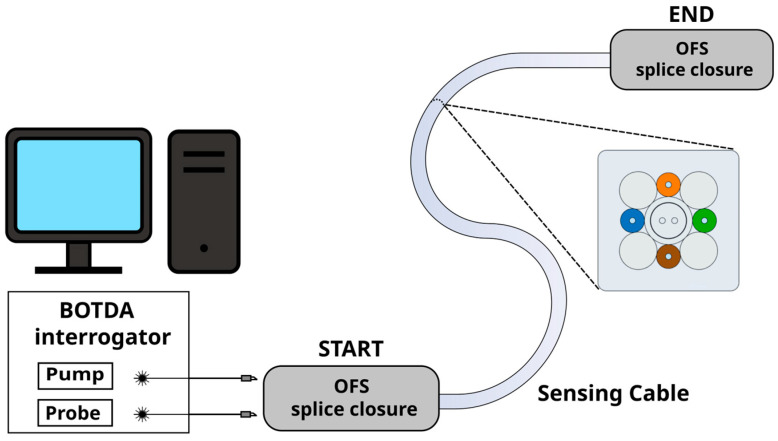
Schematic of the experimental setup, showing the disposition of the interrogating unit, the sensing cable (along with its cross-section layout), and both its starting and end points. At each extremity, the sensing fibers are spliced, and the joints are protected inside dedicated splice closures.

Experimental data were acquired in the ISMN-CNR laboratories in Bologna using the OZ Optics Foresight™ DSTS [42] as an interrogator system. The schematic of the experimental setup is shown in Figure 7.

**Figure 8:**
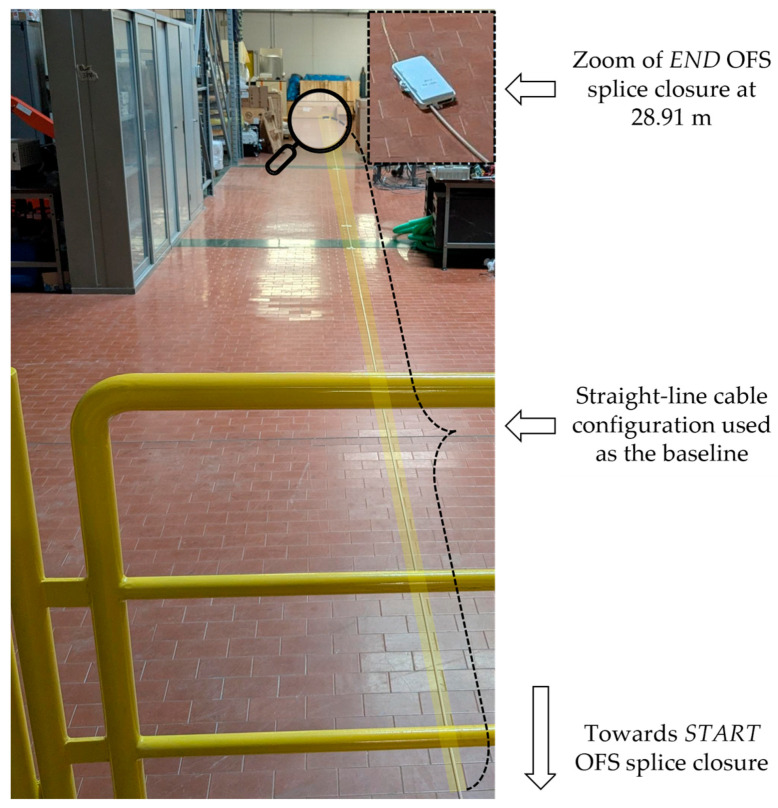
Shape sensing cable during the baseline acquisition. In order to account for possible sources of residual strain in the sensing cores, a baseline is obtained by measuring their strain while the cable is kept straight.

When the shape of the sensing cable changes, the resulting deformation induces variations in the strain measured by the fiber cores with respect to a baseline configuration. In this study, the baseline is obtained by laying the cable in a straight line on the laboratory floor (Figure 8).

The procedure is repeated four times, and a master baseline is computed as the average of the corresponding strain profiles, thereby improving the signal-to-noise ratio. The experimental parameters setup can be summarized in Table 2.

The pulse width of 5 ns translates to a spatial resolution of 0.5 m. The length of the fiber must be selected such that all the fiber segment lengths are counted, the patch cords connecting the interrogator with the OFS splice enclosure at the shape sensing cable starting point, the splicing connections are counted, and a safety margin is also counted. This resulted in the choice of analyzing a total fiber length trace of 220 m.

### 4.3. Hotspot-Based Segment Alignment

**Figure 9:**
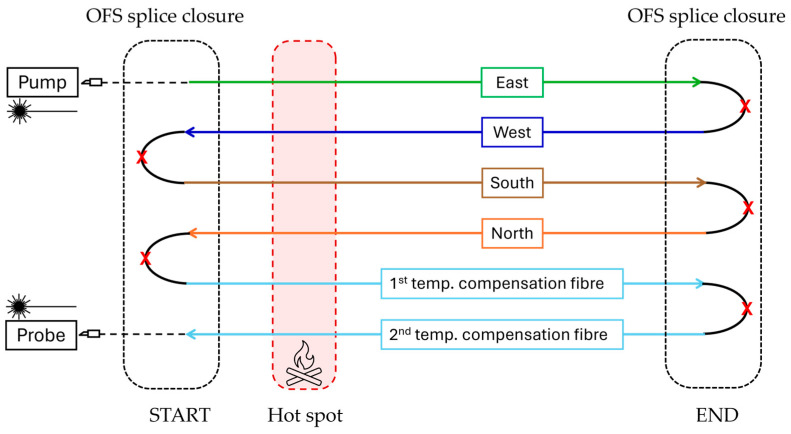
Connection scheme within the sensing cable: all sensing fibers are spliced to each other (at the points corresponding to the red “X” symbols) to form a single circuit to make BOTDA measurements possible. A hotspot is used to correlate every position in the strain measurement to its position along the sensing cable.

The hot-spot procedure constitutes the first step of the calibration process. Because the DSTS system operates with a single interrogation channel, the fiber path must be arranged so that all sensing cores are connected in series, as illustrated in Figure 9.

This configuration, however, requires that the different fiber segments be accurately synchronized in the spatial domain, as even small misalignments can lead to significant reconstruction errors.

Achieving this synchronization is non-trivial. Fusion splices located near the beginning and end of the cable reduce the signal-to-noise ratio in their vicinity. Consequently, spatial alignment must be accompanied by appropriate data truncation. This aspect must be considered during installation, as the initial and final portions of the cable may not be reliably reconstructed.

**Figure 10:**
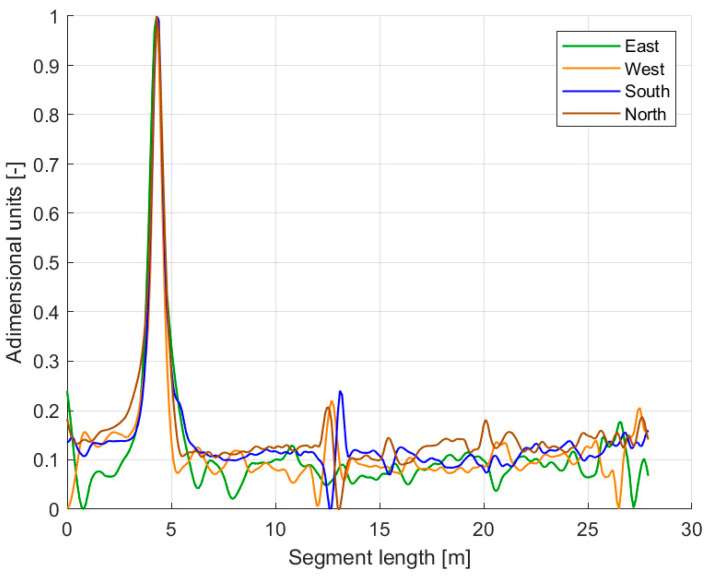
Spatial synchronization of the strain-sensing fiber segments: the BFS measurements (which have been made dimensionless and normalized) corresponding to each segment are shifted position-wise until their hot spot peaks overlap.

The hot-spot procedure consists of imposing a localized strain perturbation at a known position along the cable. The term “hot spot” originates from the common practice of generating this perturbation via localized heating, although other methods capable of producing concentrated strain inputs could alternatively be used. In this study, the hot spot was produced by a heat gun positioned 500 mm above the cable, which was laid out straight on the laboratory floor in its baseline configuration. The heating location was arbitrarily chosen at 4780 mm from the reference end of the cable. To mitigate the degradation in signal-to-noise ratio caused by splices, the original sensing interval was truncated by 500 mm at both ends of the cable. Aligning the strain peaks induced by the hot spot enables the spatial synchronization of all fiber segments (Figure 10).

Knowledge of the applied temperature is not required for this alignment. However, if the temperature is measured independently, for example, using a thermocouple, the same procedure can also be exploited to calibrate any temperature-compensation fibers included in the cable.

### 4.4. Radial Offset Calibration

As shown in Figure 6, the cable used in this study has a nominal radial offset provided by the manufacturer (Socotec Monitoring Italy S.R.L.) equal to 1.3±0.2 mm. This value represents the distance of the single-core fibers from the neutral axis of the optical fiber cable. This value might deviate from the one found in experimental campaigns. These differences can be due to technological limitations and uncertainty associated with the manufacturing process. Moreover, creep, temperature cycles, large deformations, and torsions might alter the original fiber position after a certain amount of time. All these considerations bring to the fore the importance of a proper calibration protocol for a correct radial offset estimation.

In this study, the calibration protocol consists of winding the cable around circles of known diameters (⌀ 1440 mm, ⌀ 2220 mm, ⌀ 3000 mm). Then, the optical fiber cable coils are fitted with the best radius and center coordinates of a circumference and are compared with the ground truth data.

At this point, it is possible to define an error vector whose entries are the relative errors defined by the difference between the estimated radii and the true radii for the three diameter values divided by the true radii.

Taking the norm of such a defined error vector allows us to obtain a calibration metric that can be used to find the best radial offset. Specifically, an optimization algorithm is constructed by testing 10 linearly spaced radial offset values centered on the nominal radial offset (the one given by the manufacturer). Then, by plotting the error norm against these radial offset values, one obtains a function whose minimum represents the optimal solution. By repeating this procedure iteratively and shrinking the window at each step, it is possible to compute the radial offset value that optimizes the problem.

Each calibration procedure performed with a certain circle diameter (⌀ 1440 mm, ⌀ 2220 mm, ⌀ 3000 mm) returns slightly different results due to repeatability issues, non-linear behaviors, and so on. Therefore, the best radial offset value was obtained by averaging the results obtained through the three different cases, and the best value was radial offset value for the cores was found to be $r_{c}=1.05\;mm$.

The calibration procedure also makes it possible to estimate the noise statistics of the measured strain signal; this, in turn, enables computation of the theoretical minimum detectable curvature. In our specific experimental setup, the strain standard deviation was $\sigma_{\varepsilon }=40\;\mu \varepsilon$.

For a bidimensional bending case, assuming the core lies at 90° with respect to the neutral axis, Equation (1) simplifies and yields the classical linear relation linking strain, radial offset, and curvature:

(13) $$\varepsilon \;=\kappa \cdot r_{c}$$

Using three times the standard deviation to ensure a probability of missing a curvature event lower than 1%, the theoretical minimum detectable curvature can be expressed as:

(14) $$\kappa_{min}=\frac{3\sigma_{\varepsilon }}{r_{c}}=\frac{120\mu \varepsilon }{1.05\;\mathrm{mm}}=0.11\;m^{-1}⟹r_{max}=\frac{1}{\kappa_{min}}=\frac{1}{0.11\;m^{-1}}\approx 9.1\;m$$

This value should be considered to understand if the experimental setup matches the required performance for the specific application

## 5. Case Study: S-Shaped Geometry

This section presents the case study, introducing the selected S-shaped geometry, describing how the cable is deployed and secured to the ground, and evaluating the uncertainty associated with this procedure, which affects the ground-truth reference. The second part of the section explains how sensing performance is assessed through a set of specific figures of merit and introduces a multi-criteria ranking framework used to compare the different weighting-based error-compensation algorithms, particularly useful in cases where the various figures of merit can potentially lead to contrasting evaluations.

### 5.1. Reference Geometry Definition and Uncertainty

**Figure 11:**
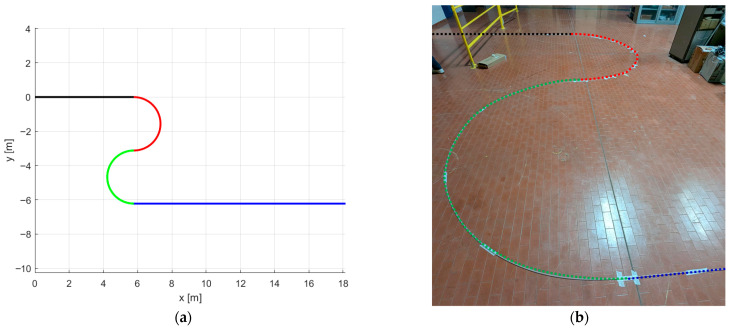
S-shape curve chosen as a case study to be reconstructed: (**a**) Ground truth curve in the x-y plane; (**b**) Experimental realization: the curve profile was drawn on the ground, and the sensing cable was made to follow it by securing it with adhesive tape.

The case study selected in this study is the S-shape (Figure 11).

The selected test geometry (Figure 11a) consists of an initial straight segment (black) of 5775 mm, followed by a half-circle (red) with a radius of 1560 mm, a second half-circle (green) with a radius of 1550 mm, and a final straight segment (blue) of 12,370 mm.

This geometry is particularly challenging for shape-sensing reconstruction because the two straight portions produce very low strain levels, resulting in a reduced signal-to-noise ratio. Moreover, the straight segments introduce an expected singularity in the torsion of the Frenet–Serret frame, further complicating reliable curve reconstruction.

Figure 11b represents the corresponding curve in the real experimental setup. The cable was fixed on the laboratory floor with adhesive tape. The square geometry of the cable made it relatively easy to ensure that the north–south-east–west configuration was preserved with respect to the global frame of reference, having the north fiber always pointing upward. To objectively define the imposed geometry, the start and end points of the straight segments, as well as the centers and radii of the circular arcs, were measured and recorded.

An operator-dependent positioning uncertainty was considered to account for small misalignments that may occur while fixing the cable to the floor. A conservative maximum deviation of ±5 mm was assumed and modeled with a rectangular distribution, yielding a type B standard uncertainty of $u_{op}=\frac{5}{\sqrt{3}}\approx 2.89$ mm.

Furthermore, since the position of the reference points along the cable was measured using a tape measure with a nominal accuracy of ±0.5 mm, assuming a rectangular distribution, the corresponding type B standard uncertainty is $u_{tm}=\frac{0.5}{\sqrt{3}}\approx 0.29$ mm.

Therefore, the combined standard uncertainty associated with the reference-point positioning is:

(15) $$u_{c}=\sqrt{u_{op}^{2}+u_{tm}^{2}}=\sqrt{{2.89^{2}+0.29}^{2}}=2.90\;\mathrm{mm}$$

If we consider a coverage factor of 2, one obtains the expanded combined uncertainty $U_{c}=5.8$ mm with a level of confidence of approximately 95%. These uncertainty values are acceptable for the ground-truth measurements, given that the total curve length is on the order of several meters.

### 5.2. Performance Evaluation

#### 5.2.1. Figures of Merit

The accuracy of each reconstruction method (m) was evaluated using several Figures of Merit (FOMs) derived from the Euclidean distance between the reconstructed curve ($r_{best,i}^{m}$) and the ground truth (GT) reference geometry. For each sensing point along the cable, the per-sample error was defined as:

(16) $$e_{i}={\left\|r_{best,i}^{m}-GT_{i}\right\|}_{2}$$

representing the pointwise three-dimensional positional deviation. From the resulting error distribution, four FOMs were computed to characterize the reconstruction accuracy: (i) the Mean Absolute Error (MAE), which quantifies the average magnitude of deviations; (ii) the Root Mean Square Error (RMSE), which emphasizes larger errors through quadratic weighting; (iii) the Standard Deviation of the Error (STD), which measures the dispersion of errors around their mean and reflects the consistency of each method; and (iv) the Maximum Error (E_max_), which identifies the worst-case deviation:

(17) $$\mathrm{MAE}\;=\;\frac{1}{n}\;\sum_{i=1}^{n}e_{i}\;$$

(18) $$\mathrm{the}\;\mathrm{RMSE}=\sqrt{\frac{1}{n}\sum_{i=1}^{n}e_{i}^{2}}$$

(19) $$\mathrm{STD}=\sqrt{\frac{1}{n-1}\sum_{i=1}^{n}{\left(e_{i}-\bar{e}\right)}^{2}}$$

(20) $$E_{max}=\underset{i}{\max}\;e_{i}\;$$

where n is the total number of sensing points, and $\bar{e}$ represents the mean error:

(21) $$\bar{e}=\frac{1}{n}\sum_{i=1}^{n}e_{i}$$

Although additional performance metrics could be employed, the selected FOMs are deemed sufficiently exhaustive to characterize the overall sensor performance. For instance, in the present case study, the end-point error [22], is not meaningful, since, due to the averaging algorithm, its value is identically zero for all the considered methods.

#### 5.2.2. Multi-Criteria Ranking

An objective comparison of the reconstruction algorithms was achieved by applying a multi-criteria performance evaluation based on the TOPSIS (Technique for Order of Preference by Similarity to Ideal Solution) framework [45], combined with entropy-based weighting [46,47,48]. The idea of weighting each FOM based on its variability across different scenarios (i.e., error compensation algorithm) derives from the concept of entropy, originated from Shannon’s information theory in 1948 [49].

Let $X=\left[x_{ml}\right]$ denote the decision matrix, where each row corresponds to one of the M reconstruction methods and each column corresponds to one of the considered error metrics, namely RMSE, MAE, STD, and $E_{max}$. Since all metrics represent error measures, lower values indicate better performance for all criteria.

First, the decision matrix was normalized column-wise to obtain a probability distribution for each metric:

(22) $$p_{ml}=\frac{x_{ml}}{\sum_{m=1}^{M}x_{ml}}$$

where $p_{ml}$ represents the normalized contribution of the method m to metric l. The information entropy associated with the metric l was then computed as:

(23) $$E_{l}=-\frac{1}{\ln M}\sum_{m=1}^{M}p_{ml}\ln\left(p_{ml}\right)$$

where M denotes the number of reconstruction methods. The normalization factor 1/lnM ensures that $0\leq E_{l}\leq 1$. The entropy-based weight $\vartheta_{l}$ for each metric was computed as:

(24) $$\vartheta_{l}=\frac{1-E_{l}}{Z}$$

where Z is the normalization constant ensuring that the weights are non-negative and sum to unity:

(25) $$Z=\sum_{l=1}^{4}\left(1-E_{l}\right)$$

Metrics characterized by lower entropy (i.e., higher information content) thus receive larger weights.

The TOPSIS approach [45] was then applied following standard steps:

(i) vector normalization of the decision matrix;
(ii) weight multiplication of the normalized matrix;
(iii) identification of positive ideal solution (PIS) and negative ideal solution (NIS) as the minimum and maximum weighted values for each metric, respectively;
(iv) for each error compensation method, computation of Euclidean distances $D_{m}^{+}$ and $D_{m}^{-}$ from the PIS and NIS, respectively;
(v) for each error compensation method, calculation of the closeness coefficient: $C_{m}$

(26) $$C_{m}=\frac{D_{m}^{-}}{D_{m}^{+}+D_{m}^{-}}$$

Different error compensation methods were finally ranked in descending order of $C_{m}$ (also denoted later as TOPSIS score), with values approaching 1 indicating superior overall performance.

This approach enables the aggregation of multiple performance indicators while avoiding the subjective assignment of criterion importance.

## 6. Results

The first part of this section compares the reconstructions obtained without applying any error-compensation strategy, by solving the Frenet–Serret equations and sweeping the TNB frame in the forward direction from the initial point and in the backward direction from the end point. The second part presents the reconstruction performance after applying the proposed error-compensation algorithm, comparing the different weighting strategies through the selected figures of merit and highlighting the benefits introduced by this approach.

### 6.1. Uncompensated Shape Reconstruction

**Figure 12:**
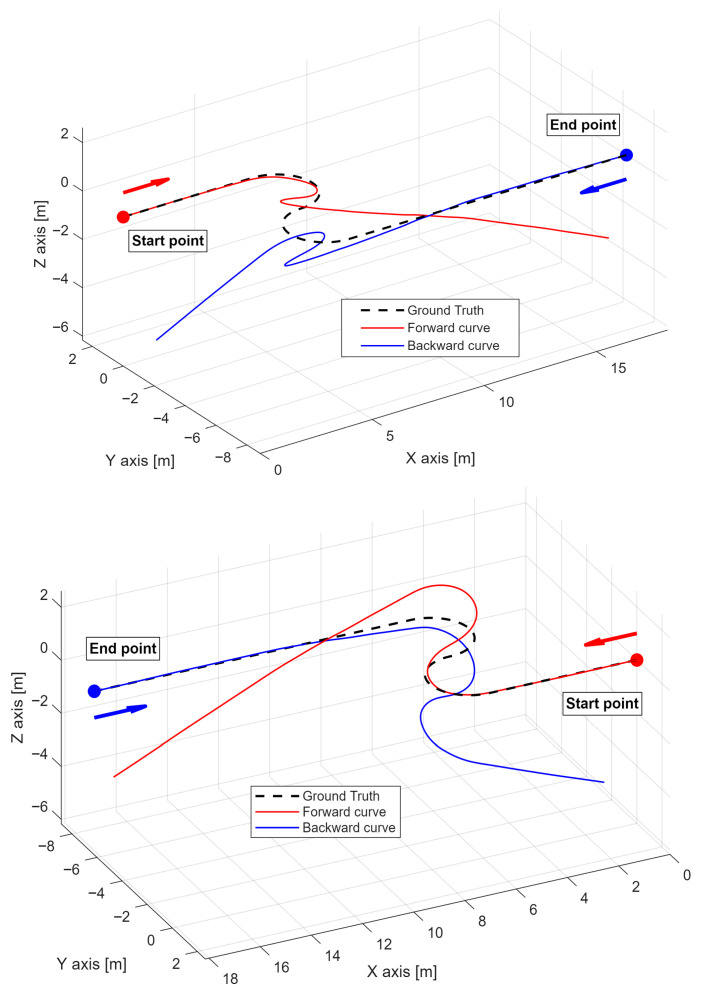
3D S-shape reconstructions obtained without applying the compensation algorithm. The (**top panel**) shows the forward reconstruction with the labeled initial point, while the (**bottom panel**) shows the backward reconstruction from the opposite endpoint. The arrows indicate the reconstruction direction: red for the forward direction and blue for the backward direction. The two views illustrate how the uncompensated forward and backward strategies diverge from the ground truth toward their respective cable ends.

Figure 12 illustrates the S-curve reconstruction performed in both forward and backward directions. The tangent vector is displayed to indicate the direction of traversal along the curve.

As shown, one of the main challenges in shape reconstruction is not the detection of curvature, since the radii of curvature are generally well estimated, but rather the handling of straight segments. In these regions, the signal-to-noise ratio is low, and error tends to accumulate in the torsion vector. This issue is not apparent within the straight segment itself because the curvature is zero, effectively masking the error. However, as soon as the curve enters a region with non-zero curvature, even if the curvature magnitude is correctly estimated, the bending angle appears incorrect due to the torsion error accumulated in the preceding straight segment.

**Figure 13:**
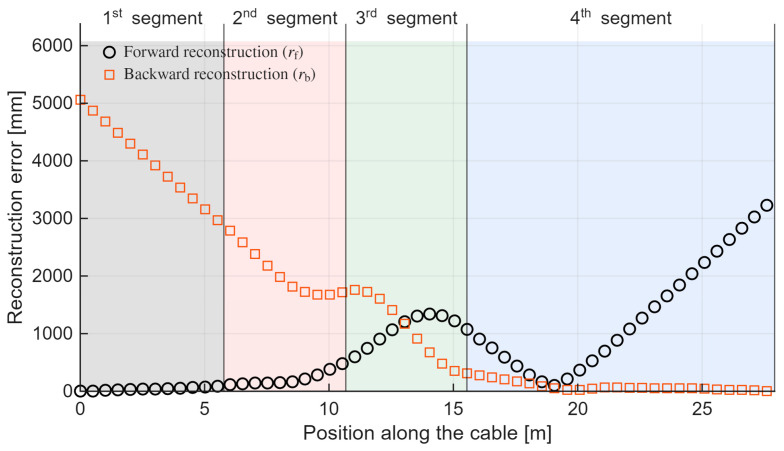
3D S-shape reconstruction error without the use of the compensation algorithm: depending on the starting point, shape reconstruction error changes, with increasing error towards the respective cable ends. The shaded colors highlight the different geometric segments of the curve, using the same color scheme introduced in Figure 11 (black for the initial straight segment, red for the first half-circle, green for the second half-circle, and blue for the final straight segment).

This concept is further illustrated in the following Figure 13, where the respective errors are plotted as a function of the curve length and highlight the corresponding geometric segments. This visualization helps identify regions of maximum error and examine whether these errors correlate with specific geometric features.

To better understand the differing error trends in the forward and backward reconstructions, it is useful to consider both panels of Figure 12 along with Figure 13.

First, the deviation is not symmetric with respect to the forward and backward directions. As noted in [17], the impact of noise in strain measurements is greater for points closer to the beginning of the curve. An error in evaluating curvature at the initial sensing points propagates along the entire curve, affecting all subsequent points. For example, in Figure 13, around 20 m, the deviation of the backward curve begins to increase linearly and more rapidly than the forward curve, influencing all downstream points. In the forward direction, the same error has a minimal effect because the point is near the end, where the deviation is already increasing rapidly.

Another factor shaping the deviation curves in Figure 13 is the sharp increases observed in the second and third segments. These distortions arise from the experimental curve design: the S-curve contains three points of discontinuity in curvature and bending angle. Due to the limited spatial resolution of the BOTDA, the corresponding strain changes are distributed over a length of at least 50 cm, resulting in transitional distortions along the curves.

This also explains why the forward curve deviation reaches zero in the fourth segment in Figure 13. As seen in Figure 12, the curve initially deviates upward relative to the ground truth, then downward, intersecting the ground truth at a single point before deviating again.

The results in Figure 12 and Figure 13 strongly emphasize the usefulness of the developed compensation algorithm, highlighting the serious issue of error accumulation, which, as stated in the introduction, makes shape reconstruction with an uncompensated BOTDA sensor impractical, particularly for an off-the-shelf model like the one used here, with deviations up to 5 m over a 28.91 m cable length. The maximum deviations from the ground truth are 3380 mm for the forward reconstruction and 5063 mm for the backward reconstruction. Beyond interrogator limitations, such as spatial resolution, other structural uncertainties may contribute, including strain transfer along the optical fiber cable [50,51].

### 6.2. Compensated Shape Reconstruction

A consistent comparison across all compensation methods and the uncompensated approach, considering both forward and backward propagation, was achieved by organizing the results into a single matrix. The matrix includes one row for each of the ten evaluated methods, along with two additional rows representing the uncompensated configurations ($r_{f}$ and $r_{b}$). The columns correspond to the four performance metrics: RMSE, MAE, Standard Error, and $E_{max}$.

The metric weights ϑ, computed from their respective entropy values as detailed in the previous section, are reported in Table 3. It can be seen that the weight values are similar for all FOMs, with only slight advantages for the maximum error and the standard deviation, indicating that no metric is significantly more descriptive than the others.

Then Table 4 shows the ranking of the results based on the derived TOPSIS score based on the closeness coefficient ($C_{m}$).

One thing that can be observed is that the bidirectional shape reconstruction always provides a significant improvement with respect to simple one-directional reconstruction. Still, the linear weighting scheme yields a comparatively poor performance with respect to other functions. This finding aligns with our previous results, which demonstrated that uncertainty does not grow linearly with distance [16]. In particular, it was shown that noise introduced near the beginning of the curve had a markedly stronger impact than noise introduced near the end, primarily due to the cumulative nature of the error propagation process [18]. The results in Table 4 suggest that, among the functions taken into consideration, the best results are obtained with a cubic weighting function. While these might indicate an overall better accuracy for this function in general, it should be considered that these results are also affected by the geometric characteristics of the system under analysis, which can further influence the specific nonlinear nature of uncertainty behavior.

Indeed, error growth is a complex issue that depends on the combined effect of all possible uncertainty sources affecting the measuring system. These include systematic strain biases due to calibration errors, Gaussian noise, SNR reduction caused by optical attenuation along the fiber, limitations imposed by the spatial resolution, and twist uncertainty [52], core position uncertainties [53], and strain-transfer effects between the cable and the host structure [54].

Depending on the trajectory to be reconstructed, interrogator technology, and sensing cable mechanical and geometrical properties, the prevalence of each of these uncertainty sources in the propagation of reconstruction error is expected to change.

**Figure 14:**
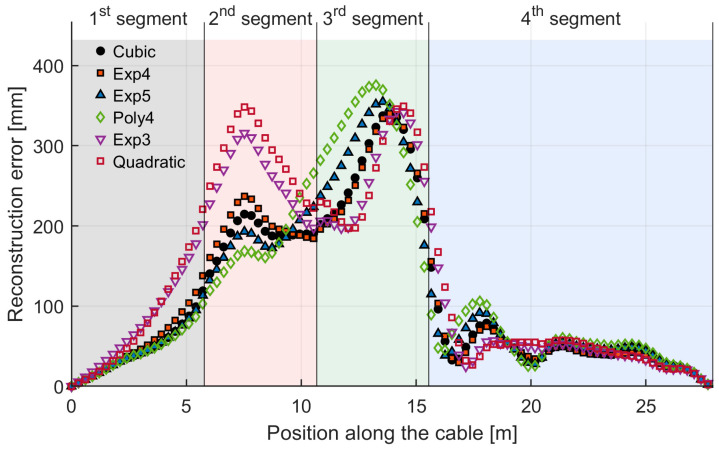
Error distribution in mm of the top 6 TOPSIS-ranked methods.

For a more specific view on the relationship between reconstruction error and distance, Figure 14 illustrates the overall error distribution along the cable length for the six best-ranked methods, as determined by the TOPSIS classification.

What can be seen is that, as expected, the error values tend to zero at the extremities because of the averaging algorithm, which incorporates the two fixed points located at the cable ends. The error reaches its peak approximately in correspondence to the midpoints of the two semicircles. Looking at the trends along the cable, all cost functions provide similar results, with peaks in the 2nd and 3rd segment and a smaller one at the beginning of the 4th. In particular, both polynomial cost functions with higher exponents (Cubic, Poly4) and exponential functions with higher factors (Exp 4, Exp5) tend to lower the peak in the 2nd segment, while not doing the same for the 3rd one. This can be explained by looking at Figure 12 and noting that in the 2nd segment, the error of the forward curve is lower compared to the backwards one, while in the 3rd one, they are both higher.

**Figure 15:**
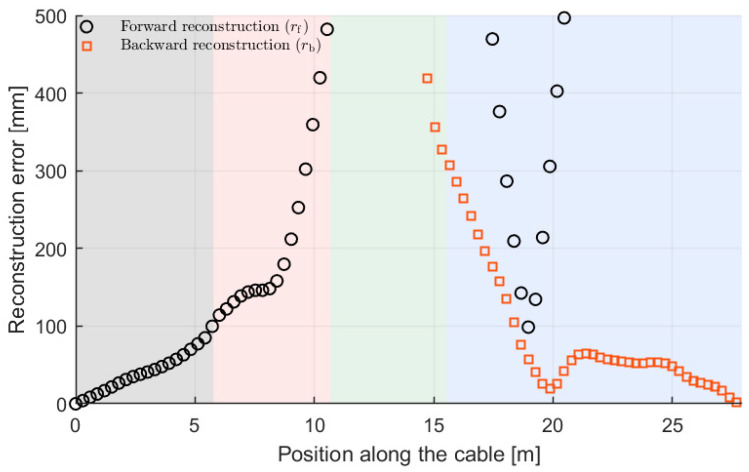
Zoom-in on the Y-axis of the results in Figure 13, showing the deviations at the initial parts of both the forward and backward curves.

To explain the specific behavior of the curve in Figure 14, particularly in the 1st and 4th segments, we consider that near the beginning of the sensing cable, the reconstructed curve $r_{best}$ largely resembles the forward curve $r_{f}$, due to the weighting function favoring it. Conversely, near the end of the cable $r_{best}$ largely resembles the backward curve $r_{b}$. Figure 15 presents the same results as Figure 13 but with a greater Y-axis zoom to focus on errors at the beginning (for $r_{f}$) and end (for $r_{b}$) of the sensing cable. Comparing Figure 14 and Figure 15 confirms that for all weighting functions, the error behavior for $r_{best}$ in the 1st segment aligns with the error for $r_{f}$ while in the 4th segment it aligns with the error for $r_{b}$ as expected.

**Figure 16:**
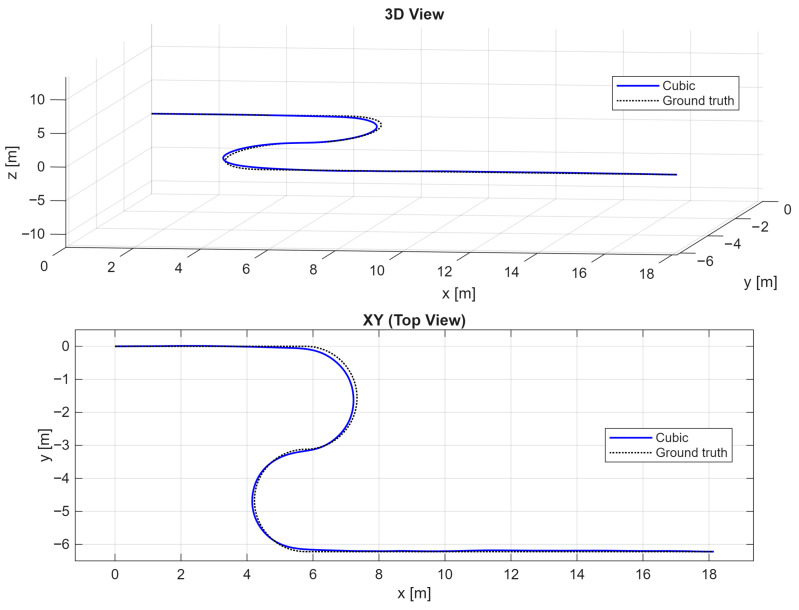
3D S-shape reconstruction (**top**) and its relative projection on the XY plane (**bottom**). The black-dotted line represents the experimental ground truth, whereas the blue solid line represents the reconstructed shape with BOTDA.

The result of the shape reconstruction obtained with the best (Cubic) compensation algorithm is shown in Figure 16.

Table 5 then reports the percentage improvements achieved by the Cubic method with respect to both forward and backward propagation:

## 7. Discussion

### 7.1. Core Contribution: Endpoint-Constrained Averaging Strategy

As was seen in Section 6, error reductions in terms of FOMs were always above 86%, showing how incorporating known end-point positions greatly improved reconstruction accuracy and mitigated spurious out-of-plane deviations due to low spatial and strain resolutions. The averaging process combines forward and backward reconstructions through weighted aggregation, improving robustness without increasing system complexity or computational burden, outside of repeating the shape reconstruction process twice.

This strategy is, of course, particularly well-suited to BOTDA-based configurations, including conventional BOTDA, BOFDA, and DPP-BOTDA systems, where sensing cables typically terminate in accessible splice enclosures. Nevertheless, it can also be applied to shape sensing applications based on other OFSs technologies, and still provide beneficial effects, which are expected to vary depending on the number of sensing points and measurement accuracy.

Of course, this method can also be easily extended to scenarios with multiple reference points by treating each segment as an independent reconstruction problem.

### 7.2. Calibration Sensitivity and Practical Limitations

The calibration procedure for radial offset tuning proved to be critical for this application. This aspect is often overlooked in multicore fibers but becomes essential in sensing cables composed of fiber bundles, where manufacturing limitations can impair accuracy along the cable length. Our approach assumes that the fibers maintain a constant distance from the neutral axis and that the cable behaves symmetrically. These assumptions may not hold in practice. A more sophisticated calibration strategy would be required to address these limitations, representing an interesting avenue for future research. Additional factors may be associated with the strain transfer phenomenon, which is typically investigated in the context of strain monitoring, where a fiber or sensing cable is bonded to a host structure [50]. In the case of shape-sensing cables, however, a dedicated analysis is required because the strain source is no longer the host structure but rather the bending and torsion of the cable itself [55].

### 7.3. Implications, Replicability, and Future Directions

The methodology proposed in this study is designed to be easily replicable, prioritizing best practices and algorithmic strategies rather than leveraging the most advanced technologies available. While more sophisticated BOTDA variants, such as DPP-BOTDA, or alternative approaches could enhance shape-sensing performance, this was not the primary objective of our work. Instead, we aimed to demonstrate that reliable results can be achieved through careful calibration and algorithmic design without resorting to complex or costly solutions.

It should be noted that no squelch algorithm or additional filtering procedures were applied to the raw strain data in this study. Future investigations could benefit from incorporating tailored signal-processing techniques that account for both the geometric and mechanical properties of the sensing cable. Such approaches might include adaptive thresholds on maximum allowable curvature or torsion, which would flag conditions potentially leading to cable damage or mechanical failure, thereby enhancing the reliability and safety of the sensing system.

Furthermore, it would be of interest to investigate the effect that this approach might have on twist suppression by leveraging not only the coordinates but also the positional and local Frenet–Serret frame orientation data. Fiber twist remains a persistent challenge in shape sensing [56,57,58]. Although our averaging algorithm does not explicitly address twist, it improves accuracy without introducing additional complexity. Future research could explore incorporating intermediate constraints, such as enforcing specific bending angles at predefined locations, to further reduce uncertainty and enhance twist control.

## 8. Conclusions

In this work, we have for the first time tackled the issue of error build-up in shape sensing applications and presented a simple yet effective method to suppress it when both the start and end positions of the fibers are known. This was achieved by performing shape reconstruction in both forward and backwards direction, and then combining them in a weighted sum, favoring the one that was closest to its origin. We have experimentally implemented this method to perform shape reconstruction of a 30 m sensing cable deployed along an S-curve, employing a BOTDA sensor. Although the cable was positioned manually, the associated operator-dependent and measurement uncertainties are negligible when compared with the geometry-reconstruction accuracy achieved in this study and are also fully consistent with the spatial resolution of the BOTDA interrogation scheme. For reference, the lowest RMSE obtained with the cubic weighting was 145.4 mm over a cable length of 28.91 m, whereas the combined standard uncertainty associated with the reference-point positioning (coverage factor k = 2, confidence level ≈ 95%) is Uc = 5.8 mm.

As we performed the reconstruction, we also tested different weighting methods for our approach, testing various polynomial and exponential functions and comparing them using an entropy-based TOPSIS score resulting from FOMs derived from the deviation of the reconstructed curve from the ground truth. We found that the optimal weight function depended on the cube of the distance from the origin. With this weight function, our method improved shape reconstruction by more than 86% in each FOM with respect to the cases where it was not used, reducing the deviation from several meters to below 40 cm.

To provide a quantitative comparison between the performance presented in this work and that of previously developed shape sensing applications, we see that without our approach, we achieve a maximum reconstruction error of at best 3380 over 28.9 m. Employing our approach, the maximum error becomes 340 mm over the same length. If we use the ratio as a metric of accuracy, we get a maximum deviation of 11.7% without compensation, and 1.18% with our compensation. Comparing this latter result with other previously mentioned examples, the relative error is lower than the ones reported in [19] (around 2%) or [22] (around 6%). If we compare it with other BOTDA-based shape sensors, it is in line with [30] (1%, but on only 2D reconstruction) and [32] (1.2%). Those works employed DPP-BOTDA, with a spatial resolution of 5 cm over a length of 1.5 m [30], or 2 cm in [32] over a length of 25 cm. On the other hand, we employed an off-the-shelf standard BOTDA sensor with a much more limited spatial resolution of 50 cm over a much greater length of 28.9 m.

Thanks to our method, we were able to reconstruct a curve with an off-the-shelf version of a sensor type that has, so far, been ill-suited for shape sensing due to its low spatial resolution. This achievement indicates that our method could prove greatly beneficial for situations where measurement noise and a large number of sensing points would cause accuracy to degrade.

It should be emphasized that this problem has received little attention to date, primarily due to two factors: first, most shape sensing applications so far involved a limited number of sensing points due to short measurement ranges or the use of point-like sensors (like FBGs). Second, an actual characterization of this phenomenon requires a statistical evaluation that can only be achieved by performing the same measurement a large number of times, which is mostly feasible in numerical simulations.

While the estimation of the optimal weight function has so far been obtained through direct comparison, a more accurate and general analysis of how measurement error propagates to the 3D curve reconstruction will no doubt provide insight regarding the optimal function and how our approach will affect different curves. These considerations suggest the need for further investigations to determine whether the observed trend is specific to the experimental and numerical conditions adopted here, or whether it reflects a more general underlying mechanism of error growth in Frenet–Serret–based fiber-shape reconstruction.

## Figures and Tables

**Table 1:** Relationship between the parameters α and k.

k	1	2	3	4	5
α	0	2.1972	3.8918	5.4161	6.8659

**Table 2:** Setup parameters used in the experimental campaign.

Base Set-Up	Fiber	Scan
Channel = CH1	Fiber Type = SMF28e	Start Frequency = 10,219.52 MHz
Fiber Length = 220 m	Calibration Temperature = 21 °C	End Frequency = 11,527.68 MHz
Pulse Width = 5 ns	Brillouin Frequency = 10,850 MHz	Frequency Step = 4.48 MHz
Spatial Step = 0.1 m	Coefficient of Strain ($C_{\varepsilon }$) = 18.915 με/MHz	Input Range = 1 V
Average No. = 64,000	Coefficient of Temperature ($C_{T}$) = 0.9765 °C/MHz	Process Method = General Analyze *

* Reconstruction of the Brillouin Gain Spectrum with the highest possible accuracy.

**Table 3:** Metrics weights (entropy analysis).

	E	1 − E	ϑ
RMSE	0.7857	0.2143	0.2424
MAE	0.8016	0.1984	0.2244
STD	0.7635	0.2365	0.2676
$E_{max}$	0.7653	0.2347	0.2655

**Table 4:** Methods ranking.

	RMSE [m]	MAE [m]	STD [m]	$E_{max}$ [m]	TOPSIS Score ( $C_{m}$) [-]
Cubic	0.1454	0.1092	0.0963	0.3410	0.9999
Exp4	0.1480	0.1113	0.0978	0.3400	0.9989
Exp5	0.1502	0.1115	0.1008	0.3551	0.9972
Poly4	0.1581	0.1157	0.1080	0.3758	0.9929
Exp3	0.1664	0.1275	0.1071	0.3436	0.9911
Quadratic	0.1768	0.1345	0.1149	0.3494	0.9866
Poly5	0.1806	0.1270	0.1287	0.4546	0.9793
Exp2	0.2217	0.1790	0.1310	0.4467	0.9656
Linear	0.3476	0.2939	0.1861	0.6629	0.9079
Exp1	1.0614	0.8664	0.6143	2.1213	0.5695
$r_{f}$	1.2084	0.8298	0.8801	3.3797	0.4286
$r_{b}$	2.0947	1.4090	1.5527	5.0630	0

**Table 5:** Percentage gain of the best-ranked TOPSIS method vs. the uncompensated reconstructions.

	$\mathrm{Cubic}\;\mathrm{vs}.\;r_{f}$	$\mathrm{Cubic}\;\mathrm{vs}.\;r_{b}$
RMSE	87.96%	93.06%
MAE	86.85%	92.25%
STD	89.06%	93.80%
Emax	89.91%	93.26%

## Data Availability

The raw data supporting the conclusions of this article will be made available by the authors on request.

## Abbreviations

The following abbreviations are used in this manuscript (listed in alphabetical order):

BFS	Brillouin Frequency Shift
BGS	Brillouin Gain Spectrum
BOTDA	Brillouin Optical Time Domain Analysis
DC	Directional Coupler
DFB	Distributed Feedback
DPP	Differential Pulse-Pair
DSTS	Distributed Strain and Temperature Sensor
EDFA	Erbium Doped Fiber Amplifier
Emax	Maximum Error
FBG	Fiber Bragg Grating
FOMs	Figures of Merit
FUT	Fiber Under Test
GNSS	Global Navigation Satellite System
GRP	Glass-Reinforced Plastic
GPS	Global Positioning System
GT	Ground Truth
MAE	Mean Absolute Error
MZM	Mach-Zehnder Modulator
NIS	Negative Ideal Solution
OC1	Optical Circulator
OFDR	Optical Frequency Domain Reflectometry
OFS	Optical Fiber Sensors
PA	Polyamide
PD	Photodetector
PIS	Positive Ideal Solution
PTB	Plastic Tube Buffer
RMF	Rotation-Minimizing Frames
RMSE	Root Mean Square Error
SBS	Spontaneous Brillouin Scattering
SHM	Structural Health Monitoring
SOA	Semiconductor Optical Amplifier
STD	Standard Deviation of the Error
TNB	Tangent, Normal, and Binormal Vectors
TOPSIS	Technique for Order of Preference by Similarity to Ideal Solution
